# The Role of Epigenetic Modifications in Neurotoxicity Induced by Neonatal General Anesthesia

**DOI:** 10.3389/fnmol.2022.877263

**Published:** 2022-04-27

**Authors:** Lin-Hui Ma, Jing Yan, Xin-Hao Jiao, Cheng-Hua Zhou, Yu-Qing Wu

**Affiliations:** ^1^Jiangsu Province Key Laboratory of Anesthesiology, Xuzhou Medical University, Xuzhou, China; ^2^NMPA Key Laboratory for Research and Evaluation of Narcotic and Psychotropic Drugs, Xuzhou Medical University, Xuzhou, China; ^3^Jiangsu Key Laboratory of New Drug Research and Clinical Pharmacy, Xuzhou Medical University, Xuzhou, China

**Keywords:** epigenetics, neonatal general anesthesia, DNA methylation, histone modifications, non-coding RNAs, RNA methylation

## Abstract

It has been widely demonstrated by numerous preclinical studies and clinical trials that the neonates receiving repeated or long-time general anesthesia (GA) could develop prolonged cognitive dysfunction. However, the definite mechanism remains largely unknown. Epigenetics, which is defined as heritable alterations in gene expression that are not a result of alteration of DNA sequence, includes DNA methylation, histone post-translational modifications, non-coding RNAs (ncRNAs), and RNA methylation. In recent years, the role of epigenetic modifications in neonatal GA-induced neurotoxicity has been widely explored and reported. In this review, we discuss and conclude the epigenetic mechanisms involving in the process of neonatal anesthesia-induced cognitive dysfunction. Also, we analyze the wide prospects of epigenetics in this field and its possibility to work as treatment target.

## Introduction

Recently, a number of preclinical studies and clinical trials have demonstrated that the general anesthesia (GA) during the neonatal period may influence brain development (Jevtovic-Todorovic et al., 2003; Warner et al., 2018; Kletecka et al., 2019). Although GA exists in this risk, some neonates have to undergo giant surgeries under GA *in utero* or after birth to treat congenital diseases or deal with delivery accidents. With the development of medicine, more and more neonates will face this dilemma, in the United States alone, more than six million children undergo surgery and anesthesia every year (Wilder et al., 2009). So, it is important and necessary to explore the mechanisms behind the anesthesia-induced neurotoxicity. Most of the studies revealed the mechanisms including acute neuron apoptosis, deficient of synaptic plasticity and neuroinflammation in the past years (Bosnjak et al., 2016; Baud and Saint-Faust, 2019). Recently, some articles also reported the changes of gene expression may take part in the cognitive dysfunction induced by the repeated anesthesia exposures (Culley et al., 2006; Pan et al., 2011), so whether the epigenetic modifications, the gene expression regulators, play an important role in this field has aroused much interest from researchers and been explored in the past decade (Peixoto et al., 2020).

Epigenetics is defined as the heritable alterations in gene expression that are not a result of alteration of DNA sequence (Zhang et al., 2020). Epigenetic modifications work as key regulators that dynamically control the outcome of gene expression in all three components of the central dogma. In the cell nucleus, 146 bp sections of DNA are coiled around an octamer of histone proteins, containing histone H2A, H2B, H3, and H4 histone, forming nucleosome. The adjacent nucleosomes join with each other *via* the histone H1 to form chromatin, which can switch between heterochromatin and euchromatin where epigenetics takes part (Zovkic et al., 2013). Generally, the epigenetic modifications include DNA methylation, post-translational modifications of histones, non-coding RNAs (ncRNAs), and RNA methylation; the first two take part in transcriptional events, while ncRNAs and RNA methylation mainly affect the post-transcriptional gene expression and also directly affect the protein production (Fu et al., 2014). Interestingly, these epigenetic modifications are not isolated but interfere with each other (Mateen et al., 2017). Many studies have illustrated their roles in learning and memory formation and conversely, memory loss (Zovkic et al., 2013; Jeffries, 2020), and also some scientist focused on the effects of epigenetics on neurotoxicity induced by neonatal GA recently.

## The DNA Methylation

### Process of DNA Methylation

The DNA methylation is one of the typical epigenetic changes, which occurs at cysteine bases, converting which to 5′-methylcytosine. Cytosine methylation occurs in cytosine–phosphate–guanine (CpG) dinucleotides within the 5′-regulatory regions of the genes. This process is catalyzed by the DNA methyltransferases (DNMTs) family, including DNMT-1, DNMT-3A, and DNMT-3B (Levenson et al., 2006; Chestnut et al., 2011). These subgroups show the obvious difference in their expression distribution and functions. The DNMT-3A/B can be thought as the *de novo* methyltransferases, which transfer the methyl to the unmodified DNA, while the DNMT-1 maintains the DNA methylation status (Mateen et al., 2017). The DNA methylation takes part in the regulation of chromatin structure and the transcription rate of genes. Generally, it is considered to turn the chromatin into the closed, highly compacted state restrictive to the transcription, resulting in the gene silencing. Interestingly, when exploring the DNA methylation changes in the experience-induced events including neuronal activity and learning and memory process, the researchers find these changes are the gene-specific and bidirectional, with some genes exhibiting increased DNA methylation while others decreasing (Miller and Sweatt, 2007; Guo et al., 2011). It is considered that the balance between the DNA methylation of memory activators and memory suppressors is more important (Zovkic et al., 2013).

### The DNA Methylation Changes Induced by Neonatal General Anesthesia

During the past decade, some studies demonstrated the role of DNA methylation in the neurotoxicity induced by the neonatal GA (Ju et al., 2016; Wu et al., 2016; Fan et al., 2021). It has been reported that the neonatal sevoflurane exposure increased the mRNA and protein levels of DNMT-3A and DNMT-3B, which upregulated the methylation level of brain-derived neurotrophic factor (BDNF) and Reelin promoter regions. These methylation changes induced the decrease of the plasticity-related proteins, BDNF, and Reelin, which led to the synaptic plasticity deficiency and cognitive impairments as a result (Ju et al., 2016). Another recent study focused on the DNA methylation in the isoflurane induced neurotoxicity (Wu et al., 2016). On postnatal 65 days, it is said that it is the increased expression of DNMT-1 and its occupancy on the promoter region of *Bdnf* exon IV in the hippocampal CA1 region of the rats that increase the methylation of 5′-cytosine in the promoter region of *Bdnf* exon IV, so the mRNA and protein levels of BDNF witnessed an obvious decrease in the rats with neonatal isoflurane exposure. To compare these two with each other, interestingly, the different anesthetics can induce the DNA hypermethylation of different genes through different DNMT subgroups at different time. Ju et al. (2016) used the DNMTs inhibitor 5-aza-2-deoxycytidine (5-AZA) to successfully reverse this sevoflurane induced abnormalities and Wu et al. (2016) chose the environmental enrichment to reverse, both of which reveal that this kind of epigenetic regulation can be chose as a target to prevent or treat the anesthesia induced neurotoxicity. To sum up, the neonatal GA exposure can lead to DNA hypermethylation of some of genes through the change of DNMT and then induce the neurotoxicity.

## Post-Translational Modifications of Histones

Histones can receive many modifications, including acetylation, methylation, and phosphorylation (Sanchez Mde and Gutierrez, 2009). Since the unmodified histone has the positive charge, these modifications alter the charge on the histones and then change the interactions between histones and DNA, which is negatively charged (Mühlbacher et al., 2006). These modifications are also important regulatory methods that regulate chromatin structure and the transcription rate of genes (Zovkic et al., 2013).

### Histone Acetylation

Histone acetylation is the most-widely explored and best-understood epigenetic modification, which is the addition of acetyl groups to the lysine residues on *N*-terminal histone tails catalyzed by histone acetyltransferases (HATs), which keep balance with histone deacetylases (HDACs). The HDACs in mammals are divided into four subfamilies, classes I, II, III, and IV. Classes I, IIa, IIb, and IV belong to the zinc-dependent HDACs and class III belongs to the NAD^+^-dependent HDACs named sirtuins (Berndsen and Denu, 2008). With the addition of negative charge on the acetyl group, histone acetylation decreases the affinity of histones to DNA and turns the chromatin into the open state amenable to translation. Through the regulation of target genes such as *Bdnf*, many recent studies demonstrated that histone acetylation takes part in the formation of long-term memory and conversely, the memory loss (Levenson et al., 2004).

A number of studies reported that the neonatal GA results in the decrease of histone acetylation, inducing the cognitive dysfunction as a result, which can put the blame on the imbalance of HDACs and HATs, the increase of HDACs or the decrease of HATs, either the quantity or the activity. Wu et al. (2016) reported a significant increase in the expression of HDAC2 along with an increased occupancy of HDAC2 on the promoter region of *Bdnf* exon IV in hippocampus of rats neonatally exposed to isoflurane, which as a result, revealed a decreased histone H3 acetylation in the promoter region of *Bdnf* exon IV as well. Zhong et al. (2015) reported that the neurocognitive dysfunction induced by repeated exposure to isoflurane in neonatal mice is related to the dysregulation of hippocampal histone acetylation which only affected the specific residue H4K12. To compare with these two studies, we can speculate that the histone acetylation modification has species difference. Jia et al. (2016) explored the neurotoxicity induced by sevoflurane exposure in neonatal rats. In this study, researchers reported that the elevated levels of HDAC3 and HDAC8 but no significant change in the levels of HDAC1 and HDAC2 in the hippocampus of rats neonatally exposed to sevoflurane, and they also found the decreased levels of acetyl-H3K9, acetyl-H3K14, acetyl-H4, acetyl-H4K5, and acetyl-H4K12 in the hippocampal CA1 region of the sevoflurane-exposed rats. The data upon were all tested on the postnatal 33 days, 1 h after the fear conditioning test. When explored the GA effects on the maternal-offspring module, the researchers indicated that infusion with propofol in maternal rats on gestational day-7 leads to impairment of learning and memory in off-spring, increased the protein levels of HDAC2, decreased the levels of acetylated H3K14 and H4K12 (Lin et al., 2018b). The different results of the different anesthetics suggest that the histone acetylation may have the drug specificity.

Apart from the histone hypoacetylation regulated by the increase of HDACs, some studies also reported the other way which decreases the acetylation of histones that plays an important role in the neonatal anesthesia induced cognitive dysfunction. The cAMP response element-binding (CREB) protein is a transcription factor that regulates the expression of genes related with learning and memory process. Phosphorylation of CREB is recognized as active state which is a marker of memory processing in the hippocampus for spatial learning (Mizuno et al., 2002). It was reported that propofol anesthesia during pregnancy resulted in decrease in phosphor-CREB protein in offspring rats’ hippocampus (Lin et al., 2018b). In the hippocampus of rats 24 h after exposing to a sedative dose of midazolam followed by combined nitrous oxide and isoflurane anesthesia for 6 h at postnatal 6 days, the researchers noticed the fragmentation of the CREB-binding protein (CBP), which has the HAT activity that transfers the acetyl group on the lysine residues of histone tails (Dalla Massara et al., 2016). The fragmentation of CBP led to the significant decrease of its HAT enzyme activity, the obvious reduction of the expression of acetylated H3 at *c-Fos* and *Bdnf* promoter and CREB transcription start sites and further, the decrease in the mRNA and proteins of c-Fos and BDNF in the hippocampus. This post-translational regulation points out a novel way to explore the mechanism of GA induced histone acetylation changes. Also, they show us a new method to prevent or treat the neonatal GA-induced neurotoxicity. In some of the studies, the researchers have successfully attenuated the GA-induced dysregulation of histone acetylation in the hippocampus and the neurocognitive impairment through the treatment of the HDACs inhibitor trichostatin A (TSA) and sodium butyrate (Zhong et al., 2015; Jia et al., 2016). However, no chemical interventions have been used to increase the HAT enzyme activity so to reverse the GA-induced hypoacetylation of histones or ameliorate the cognitive dysfunction, and maybe this will be explored further and testified in the near future.

### Histone Methylation

Histone methylation is another covalent post-translational modification but have a different positive or negative effects on gene transcription depending on the residue modified and the number of methyl groups that added (Berger, 2007). Generally, dimethylation of H3K9 correlates with transcriptional silencing and trimethylation of H3K4 is linked to active transcription. Recent studies focused on the function of histone methylation in the process of long-term memory storage. The researchers reported both trimethylation of H3K4 and dimethylation of H3K9 in hippocampus are in the process of memory formation, suggesting both increases in gene expression and gene repression exist during this process (Gupta et al., 2010). Nowadays, the role of histone methylation in the neonatal GA exposure-induced neurotoxicity is largely unknown. However, a recent study explored its association with impaired cognition in middle-aged mice experiencing anesthesia and surgery. Wu et al. (2019) indicated that the expression of H3K9me3 and its binding to the Bdnf exon IV promoter increased significantly in mice 24 h after the surgery under GA, and also the expression of BDNF was down-regulated. Although the module of this study was post-operation middle-aged mice, considering the bidirectional regulation of this modification and the lack of deep investigation, this mechanism gives us a novel insight into the neonatal anesthesia neurotoxicity as well.

## Non-Coding RNA

The ncRNAs are always described as RNAs that do not encode protein, which include microRNAs (miRNAs), long ncRNAs (lncRNAs), circular RNAs (circRNAs), and natural antisense transcripts (NATs) (Wu and Kuo, 2020). This kind of RNAs regulate various levels of gene expression physiologically and pathologically by modulating transcription and post-translational modifications (Mattick and Makunin, 2006; Chung et al., 2011; Sang et al., 2018). Many reports have demonstrated the important functional roles of ncRNAs in the pathogenesis of neurodegenerative diseases (Chung et al., 2011; Sang et al., 2018; Wu and Kuo, 2020).

### The miRNAs Expression Changes Induced by Neonatal General Anesthesia

One class of the ncRNA that has been widely studied in recent years is miRNAs which are endogenous approximately 22-nucleotide-long RNAs that play as post-transcriptional gene regulatory molecules in multicellular organisms (Bartel, 2004). Recent advanced studies uncovered numerous molecular mechanisms of miRNA, among which, translation inhibition or degradation of target messenger RNAs (mRNAs) is the most widely and mainly explored mechanism (Correia de Sousa et al., 2019). Considering the regulatory function of miRNAs, it has been clearly demonstrated that miRNAs have a crucial impact on cognitive function and take part in the development of several neuropsychiatric disorders, including schizophrenia, intellectual disability, and autism spectrum disorders (Xu et al., 2012).

**FIGURE 1:**
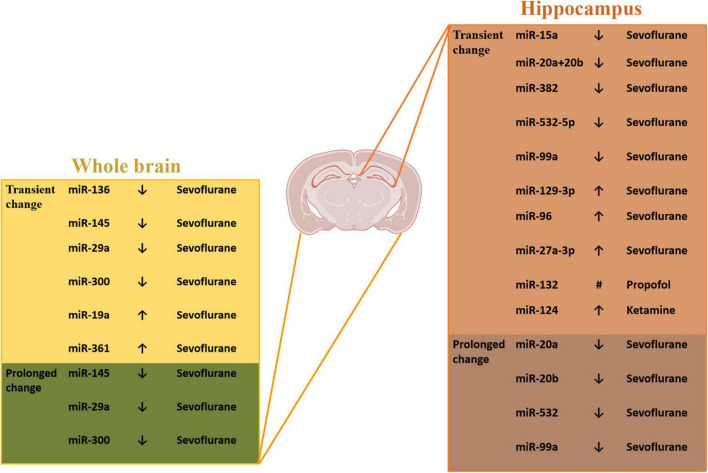
The miRNAs expression changes induced by neonatal GA. ^#^Different expression at different time points - decrease at 4, 8, and 48 h after the propofol treatment and at 14, 28, and 60 days of age; increase at 16 and 24 h after the propofol treatment.

In the recent years, many studies have focused on the roles of this post-transcriptional modification in the neonatal GA-induced prolong cognitive dysfunction (Figure 1). Lin et al. (2018a) carried out a comprehensive unbiased profile assay that revealed the effects of neonatal sevoflurane exposure on miRNA expression of the brain. The data shows that the neonatal sevoflurane had significant impact on the expression of specific miRNAs, and this GA-induced changes have region and time specificity. The hippocampus miRNAs have a region-specific expression pattern compared to the whole brain. On the same day of sevoflurane exposure, the differentially expressed miRNAs in the whole brain were miR-136, miR-145, miR-19a, miR-29a, miR-300, and miR-361, in the hippocampus were miR-129-3p, miR-15a, miR-20a + 20b, miR-382, miR-532-5p, and miR-99a, which showed a transient change after anesthesia. However, when analyzed the miRNA expression at adult to explore the long-lasting impact, some difference occurred. At adult, among the tissue from the whole brain, the expression of miR-145, miR-29a, and miR-300 showed the significant difference, and in the hippocampus, the changes were observed in miR-20a, miR-20b, miR-532, and miR-99a.

Some other articles have reported single change of certain miRNAs and changes of their target gene transcription induced by neonatal GA exposure. It has been demonstrated that rats inhaled sevoflurane at postnatal-seven-day exhibited notable increase expression of miR-96 which targets and negatively regulates insulin-like growth factor-1 receptor (IGF-1R), a receptor tyrosine kinase (Xu et al., 2019). The IGF-1 is a kind of neuroprotective hormone and plays an important role in learning and memory process (Deak and Sonntag, 2012). The downregulation of IGF-1 contributes cognitive dysfunction and memory impairment in aged-mice after sevoflurane anesthesia (Jiang et al., 2017). So, under the consideration of IGF1, Xu et al. (2019) also reported the neuronal apoptosis and cognitive dysfunction through this mechanism. Lv et al. (2017) found the increased expression levels of miR-27a-3p, the downregulation of PPAR-γ, a target of miR-27a-3p, and also the neuron injury *in vitro* after treatment of 4% sevoflurane. When inhibiting miR-27a-3p *in vivo*, the sevoflurane-induced neuronal apoptosis and cognitive dysfunction were reversed, which demonstrated the important role of miR-27a-3p.

Apart from the inhaled anesthetics, some scientists explored the neonatal neurotoxicity of intravenous anesthetics. Zhang et al. (2017) found that the repeated propofol anesthesia resulted in a downregulation or upregulation of miR-132 levels at different time points and the changes of mRNA and protein level of p250GAP, a prominent target for miR-132. This change of miRNA-132 resulted in a decrease in the number of dendritic spines in the hippocampus leading to learning and memory impairment of rats. In the study of Xu et al. (2015), they revealed the *in vitro* results that miR-124 was upregulated by ketamine in hippocampal organotypic culture. To investigate the function of the change of miR-124, they did the *in vivo* experiments by using the lentiviral vectors containing miR-124 antisense oligonucleotide to inhibit miR-124. The results showed that miR-124 inhibition strengthened glutamate receptors and upregulated PKC pathway after ketamine-exposure at postnatal 14 days, which reversed the hippocampal neurodegeneration and improved memory loss. Generally, many miRNAs have been demonstrated to take part in the GA-induced neurotoxicity through whether profile analysis or the focus on some certain miRNAs, and this modification can also be interfered by virus.

### The lncRNAs Expression Changes Induced by Neonatal General Anesthesia

The lncRNAs are another class of ncRNAs, recent studies have identified many paradigms that illustrate how lncRNAs function, including targeting proteins to specific genomic location to affect transcription patterns, modulating the activity of protein-binding partners and serving as precursors for small RNAs (Wilusz et al., 2009). A recent study in 2018 uncovered the effects of lncRNA metastasis-associated lung adenocarcinoma transcript 1 (MALAT1) on sevoflurane-induced neurotoxicity in developing rats (Hu et al., 2019). It demonstrated that rats anesthetized with sevoflurane show the high expression of MALAT1, the downregulation of BDNF and NGF mRNA and protein levels, the increase of neuron apoptosis and pathological structural changes in hippocampus and the impairment of spatial learning and memory ability. However, the downregulation of MALAT1 through MALAT1 small interfering RNA alleviated these changes. Although the relative studies are not so numerous, this kind of regulation has shown the relationship with apoptosis, deformation and memory dysfunction which are known as possible mechanism of neonatal neurotoxicity, the prospect of ncRNAs in the field of GA-induced neurotoxicity is wide and broaden.

## The RNA Methylation

### Process of RNA Methylation

The RNA methylation, as the most prevalent modification of RNA, has been widely explored in the past decade. The RNAs exist in some modified bases that can fine-tune the translation of target genes. In the past, it was thought that tRNA and rRNA have these bases and intensive researches have poured into them, while recently it was discovered that also mRNAs have modified bases, including m^6^A, m^5^C, and m^1^A, where m6A is the most-widely explored modification of bases (Squires et al., 2012; Fu et al., 2014; Dominissini et al., 2016). The m^6^A RNA methylation has been described as a dynamic and reverse modification which executes its function through a series of enzymes, including methyltransferases that help the addition of methyl groups, called writers, demethylases that help removal, called erasers and m^6^A binding proteins that help recognition and function, called readers. Considering the characteristic upon, it is also seen as a member of post-transcriptional epigenetic modifications. The m^6^A has been demonstrated to take part in the regulation of mRNAs, including splicing, localization and translation (Wang et al., 2014, 2015; Zhao et al., 2014). Considering this function, m^6^A also regulates many cellular processes and affects the memory formation and cortical neurogenesis (Yoon et al., 2017; Leonetti et al., 2020).

### The RNA Methylation Induced by Neonatal General Anesthesia

Nowadays, some researchers tried to focus on the role of RNA methylation modification in neonatal GA induced neurotoxicity. Zhang et al. (2021) reported that multiple sevoflurane anesthesia exposures may induce the downregulation of YTHDF1 in the prefrontal cortex of mice which is a critical reader protein of m^6^A RNA methylation reported to promote synthesis of target transcripts in the hippocampus of adult mice and thereby facilitate learning and memory (Shi et al., 2018). As a result, this change of YTHDF1 led to the decrease of m^6^A RNA methylation and protein level of the presynaptic protein synaptophysin and cognitive deficits (Zhang et al., 2021). After this experiment, to further investigate the possible function and effect of m^6^A modification on this sevoflurane-related neurotoxicity, these researchers published an article this year that revealed the changes in the m^6^A methylation profile of the prefrontal cortex of infant rhesus macaques (Chen et al., 2022). Compared with the control group, a dramatic increase in the number of m^6^A marks was observed in the sevoflurane group, and the hypermethylated m^6^A sites were enriched in the regulation of synaptic plasticity which may have impact on cortical development and learning and memory ability. Although the research that investigate the role of m^6^A methylation in neonatal GA-induced neurotoxicity is not that many, the articles before have offered some clues for the future exploration. The transient or prolonged changes of certain genes in the anesthesia process may be deeply assessed and can also be chosen as the targets for prevention or treatment of the neurotoxicity. Apart from the side effects of anesthetics for neonates, He et al. reported the role of m^6^A RNA methylation in POCD. They demonstrated that the weakened METTL3 (the methyltransferase works as “writer”) affinity contributed to the disorder of m^6^A RNA methylation in the hippocampus of POCD mice (He and Wang, 2021). Unlike regulations occurred on the “reader” level, the changes in POCD were showed to be as a result of “writer”, maybe the difference can attribute to the different model with different age point or some other deep reasons needed to be explored further.

## Interactions of Epigenetic Modifications

### Interaction Between DNA Methylation and Histone Acetylation

Unlike two sides of the same coin, the interactions of these three epigenetic modifications are close and comprehensive that still need long time to understand (Mateen et al., 2017). When the methyl groups were added on the region of DNA promoters, they could recruit histone-modifying enzymes, induce deacetylation of histone protein tails and condense the chromatin. This series of events reveal that the methylation of DNA results in the deacetylation of histones which works as a compounded inhibitory effect on transcription (Siegfried and Simon, 2010; Baylin and Jones, 2011).

### Interaction Between Histone Methylation and DNA Methylation

In contrast, the altered histone methylation can change DNA methylation. When researchers explored the function of epigenetic modification in the process of memory formation and consolidation, they reported that the increased H3K4 trimethylation can be associated with either increased DNA methylation (*Zif268*) (Gupta et al., 2010) or decreased DNA methylation (*Bdnf*) (Lubin et al., 2008) at genes that are transcriptionally activated in memory process.

### Interactions of Epigenetic Modifications Induced by Neonatal General Anesthesia

This kind of bidirectional interactions were also reported exist in the GA-induced neurotoxicity. Wu et al. (2016) have indicated the bridge molecular MeCP2 that binds to the methylated CpG sites in the promoter region and form the MeCP2-DNMT1 complex to maintain DNA methylation, at the meanwhile, MeCP2 recruits HDAC2 to remove the acetyl group from the histones (Kimura and Shiota, 2003), thereby leading to the more repressive transcription of target gene *Bdnf*, and this mechanism is involved in the isoflurane induced cognitive dysfunction. Also, some of miRNAs regulate DNA methylation through downregulating the expression of DNMT (Chen et al., 2014) and some can regulate the histone modifications through the transcription factor CREB. Gao et al. (2010) illustrated that the increase expression of miR-134 following SIRT1 (belongs to HDAC III family) deficiency results in the downregulation of CREB and BDNF, thereby impairing synaptic plasticity. It has been reported that the neonatal GA can lead to the decreased expression of SIRT1 and then resulted in the cognitive dysfunction (Tang et al., 2020, 2021), so whether the downstream of SIRT1, miRNAs take part in this GA induced change is worthy to be explored further.

## Conclusion and Prospects

**FIGURE 2:**
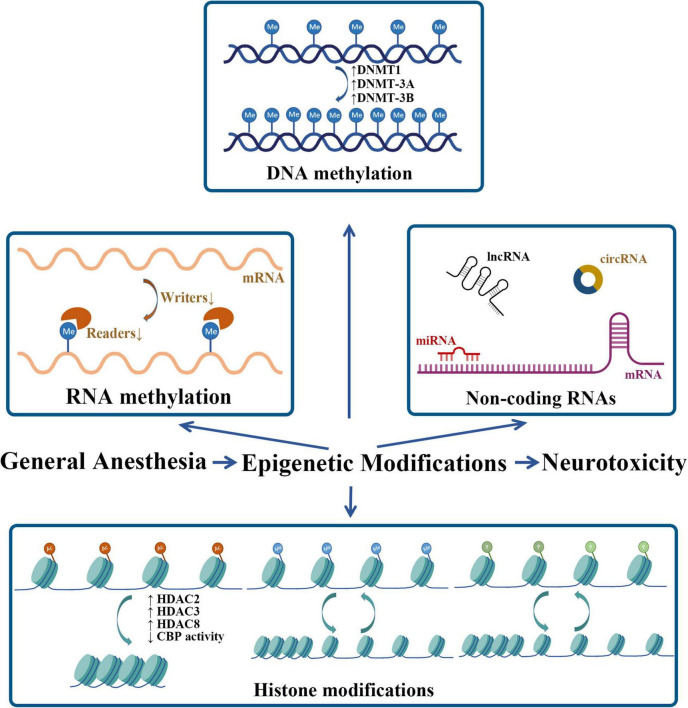
Neonatal GA exposure-induced epigenetic modifications. Me, methyl; Ac, acetyl; P, phosphate.

In this study, we summarize the role of epigenetic modifications in neurotoxicity induced by neonatal GA (Table 1 and Figure 2). Comparing with other mechanisms that have been widely reported, epigenetics is still a novel field needed to be fully explored further. Actually, the DNA methylation and the histone modifications are the two that have been investigated for a longer time; in contrast, the researches about post-translational modifications of ncRNA and RNA methylation are too few so far. Considering its broaden prospect, there are still some problems that should be solved in the future. The histone post-translational modifications in the GA-induced cognitive impairment mainly focused on histone acetylation and seldom on methylation, but other important modifications are absent, such as histone phosphorylation, which has been demonstrated to take part in the isoflurane induced oxidative stress and DNA damage (Ni et al., 2017). Since the function of ncRNAs has not been totally understood, with more and more studies focus on the potential function of ncRNAs in the future, their role in GA-induced neurotoxicity will be more clearly as well, and also, it will be likely to have a space for some other kinds of ncRNAs, such as circRNAs, which show many important biological functions by working as miRNAs or protein inhibitors (Kristensen et al., 2019). As for RNA methylation, researches in this field witnessed an outbreaking increase in the past 10 years, the reversible m^6^A methylation of mRNA was also emphasized as one of important epigenetic modifications. Related studies that explored the role of it in the GA-induced neurotoxicity recently preferred to m^6^A RNA methylation profiling analysis which gave us some clues for the future exploration. So, more studies need to be taken to furtherly testify the function and importance of DEGs in these sequencing analyses and also, their time-point changing features and species characters are also worthy of exploration, which can help these DEGs turn into prevention or treatment targets in the future. In this study, we have analyzed some interventional methods such as chemical inhibitors or virus injection that can reverse the downstream changes of epigenetic modifications. Although the animal experiments showed the obvious treatment effects, the clinical feasibility of these ways still need long time to be testified. To sum up, deep and further researches are still needed to explore the role of epigenetic modifications in this filed.

**TABLE 1:** Neonatal GA exposure-induced epigenetic modifications.

Epigenetic modifications	Expression	Distribution	Drug	Associated articles
DNA methylation DNMT-3A, DNMT-3B	↑ ↑	Hippocampus of rats	Sevoflurane	Ju et al., 2016
DNA methylation DNMT-1	↑ ↑	Hippocampus of rats	Isoflurane	Wu et al., 2016
H3 acetylation HDAC2	↓ ↑	Hippocampus of rats	Isoflurane	Wu et al., 2016
H4K12 acetylation	↓	Hippocampus of mice	Isoflurane	Zhong et al., 2015
H3K9, H3K14, H4K5, H4K12 acetylation HDAC3, HDAC8	↓ ↑	Hippocampus of rats	Sevoflurane	Jia et al., 2016
H3K14, H4K12 acetylation HDAC2	↓ ↑	Hippocampus of off-spring rats	Propofol	Lin et al., 2018b
H3 acetylation CBP activity	↓ ↓	Hippocampus of rats	Midazolam + nitrous oxide + isoflurane	Dalla Massara et al., 2016
H3K9 me3	↑	Hippocampus of mice	Surgery + isoflurane	Wu et al., 2019
miR-136, miR-145, miR-29a, miR-300 miR-19a, miR-361 miR-15a, miR-20a + 20b, miR-382, miR-532-5p, miR-99a miR-129-3p	↓ ↑ ↓ ↑	Whole brain of mice Hippocampus of mice	Sevoflurane	Lin et al., 2018a
miR-96	↑	Hippocampus of rats	Sevoflurane	Xu et al., 2019
miR-27a-3p	↑	Hippocampal of mice	Sevoflurane	Lv et al., 2017
miR-132	#	Hippocampus of rats	Propofol	Zhang et al., 2017
miR-124	↑	Hippocampus of mice	Ketamine	Xu et al., 2015
MALAT1	↑	Hippocampus of rats	Sevoflurane	Wilusz et al., 2009
m^6^A mRNA methylation of synaptophysin	↓	Prefrontal cortex of mice	Sevoflurane	Zhang et al., 2021
m^6^A mRNA methylation of FAKBP2, PAQR6, ELAVL3, NA, SAFB2, UBALD2, RAB13, RIN1	↑	Prefrontal cortex of rhesus macaques	Sevoflurane	Chen et al., 2022
m^6^A mRNA methylation of RBL1	↓			
m^6^A mRNA methylation of BDNF, SOX2, SYN1 m^6^A mRNA methylation of BACE1, IL-17A	↓ ↑	Hippocampus of mice	Sevoflurane	He and Wang, 2021

*^#^Different expression at different time points - decrease at 4, 8, and 48 h after propofol treatment and at 14, 28, and 60 days of age; increase at 16 and 24 h after the treatment.*

## Author Contributions

L-HM drafted the original manuscript. JY and X-HJ checked the cited data. C-HZ and Y-QW revised the manuscript. All authors have read and approved the final manuscript.

## Conflict of Interest

The authors declare that the research was conducted in the absence of any commercial or financial relationships that could be construed as a potential conflict of interest.

## Publisher’s Note

All claims expressed in this article are solely those of the authors and do not necessarily represent those of their affiliated organizations, or those of the publisher, the editors and the reviewers. Any product that may be evaluated in this article, or claim that may be made by its manufacturer, is not guaranteed or endorsed by the publisher.
